# A High-Efficiency CuO/CeO_2_ Catalyst for Diclofenac Degradation in Fenton-Like System

**DOI:** 10.3389/fchem.2019.00796

**Published:** 2019-11-19

**Authors:** Jia Zhu, Guangming Zhang, Guang Xian, Nan Zhang, Jinwei Li

**Affiliations:** ^1^School of Construction and Environment Engineering, Shenzhen Polytechnic, Shenzhen, China; ^2^School of Environment & Natural Resource, Renmin University of China, Beijing, China; ^3^Department of Military Installations, Army Logistics University of PLA, Chongqing, China

**Keywords:** CuO/CeO_2_, ultrasonic impregnation, Fenton-like system, OH, oxygen vacancies

## Abstract

An efficient Fenton-like catalyst CuO/CeO_2_ was synthesized using ultrasonic impregnation and used to remove diclofenac from water. The catalyst was characterized by N_2_ adsorption-desorption, SEM-EDS, XRD, HRTEM, Raman, and XPS analyses. Results showed that CuO/CeO_2_ possessed large surface area, high porosity, and fine elements dispersion. Cu was loaded in CeO_2_, which increased the oxygen vacancies. The exposed crystal face of CeO_2_ (200) was beneficial to the catalytic activity. The diclofenac removal experiment showed that there was a synergistic effect between CuO and CeO_2_, which might be caused by more oxygen vacancies generation and electronic interactions between Cu and Ce species. The experimental conditions were optimized, including pH, catalyst and H_2_O_2_ dosages, and 86.62% diclofenac removal was achieved. Diclofenac oxidation by ·OH and adsorbed oxygen species was the main mechanism for its removal in this Fenton-like system.

## Highlights

- CuO/CeO_2_ was prepared with ultrasound to remove diclofenac in Fenton-like system.
- Ultrasound made CuO/CeO_2_ had large surface area, high porosity and fine elements dispersion.
- More oxygen vacancies caused by Cu doping were in favor of the catalytic reaction.
- 86.62% diclofenac removal was achieved under the optimal conditions.
- ·OH and adsorbed oxygen species were responsible for diclofenac degradation.

## Introduction

Advanced oxidation processes (AOPs) for wastewater treatment have attracted extensive attention due to the quick and efficient pollutant removal by strong oxidizing free radicals, like hydroxyl radical (Silveira et al., 2015; Zhu et al., 2019). The most widely used AOP is Fenton reaction (Fe^2+^ activate H_2_O_2_), which is a homogeneous reaction that requires low pH (2.0–4.0) and forms large amount of iron sludge (Blanco et al., 2016). To overcome these disadvantages, heterogeneous catalysts have been adopted as the alternative (Lei et al., 2015), forming heterogeneous Fenton-like systems (Nidheesh, 2015).

The frequently used catalysts in heterogeneous Fenton-like systems are transition metal-based catalysts (Anipsitakis and Dionysiou, 2004; Bokare and Choi, 2014), due to the good catalytic activity, low cost, easily available materials, and abundant reserves. Among them, catalysts containing Cu^2+^ are in the limelight (Gu et al., 2019), because of the similar redox properties of Cu^2+^/Cu^+^ to Fe^3+^/Fe^2+^, broad pH workable range, few sludge production and easily decomposition of Cu^2+^ complexes by ·OH (Bokare and Choi, 2014; Peng et al., 2019). As one of the most common and simplest copper compounds, CuO has successfully activated H_2_O_2_ to form ·OH to remove pollutants (Sehati and Entezari, 2017). However, CuO nanoparticles tend to agglomerate in water which is unfavorable for catalytic reaction. Besides, the leached Cu ions are poisonous. Loading CuO on support is an effective way to overcome the above problems.

Some metal oxides (such as CeO_2_, Al_2_O_3_), clays, zeolites and carbon materials have been used as supports (Rao et al., 2018; Xu et al., 2018). The support increases the specific surface area of catalyst and reduces the leaching of metal ions (Du et al., 2016), which are beneficial to the adsorption property and stability of catalyst. Especially, CeO_2_ can enhance the catalytic property of catalyst for its unique structure and redox property. CeO_2_ has abundant oxygen vacancy defects and Ce^4+^/Ce^3+^ redox couple (Chong et al., 2016), so it has high oxygen storage capacity, which is beneficial for catalytic reaction.

Doping transition metal ion into CeO_2_ can create more oxygen vacancies due to the different ionic radii between Ce ion and transition metal ion, and the number of oxygen vacancies depends on the ionic radius and electrons of transition metal ions (Raj et al., 2017). Thus, the catalytic activity is improved by enhancing the oxygen storage and redox capacity (Parvas et al., 2014). The catalytic performance improvement by Cu doped into CeO_2_ has been reported before (Wang et al., 2006; Yang et al., 2009; Lin et al., 2011).

The traditional impregnation method takes a long doping time and metal species agglomerate easily, due to the small mass transfer force between CeO_2_ and the doping ions (Wang et al., 2006; Yang et al., 2009; Lin et al., 2011). Ultrasound has been used in the impregnation stage to overcome these disadvantages. Ultrasound waves induce the cavitation effect in water, which is related to the formation, growth, and rapid collapse of cavitation bubbles (Mirtamizdoust et al., 2017; Zhang et al., 2017). The ultrasonic cavitation can significantly accelerate the mass transfer velocity and provide thermal effects (Zhu and Zhang, 2008; Li et al., 2018). Therefore, ultrasound impregnation increases active components (transition metal ions) doped onto the surface of support (like CeO_2_). Meanwhile, some active components may be introduced into the structure of support under the power of ultrasound, thus the catalyst prepared by ultrasound impregnation would have better catalytic performance (Chong et al., 2016). Moreover, ultrasound can significantly shorten the preparation time of catalyst by providing faster mass transfer rate.

In this paper, CuO was doped into CeO_2_ by ultrasonic impregnation to form CuO/CeO_2_, which was then applied as a catalyst in Fenton-like process. Diclofenac, a typical pharmaceutical and emerging contaminant, was used as the target pollutant to check the activity of the catalyst. The catalyst was characterized by N_2_ adsorption-desorption, scanning electron microscope (SEM), X-ray powder diffraction (XRD), X-ray photoelectron spectra (XPS), high resolution transmission electron microscope (HRTEM), and Raman analyses. Influences of experimental parameters including pH, catalyst dosage, and H_2_O_2_ dosage on diclofenac removal were investigated. The potential reaction mechanism was proposed. The objective was to obtain a highly active Fenton-like catalyst with facial synthesis.

## Materials and Methods

### Materials

All chemicals used were analytical grade. Cu(NO_3_)_2_·4H_2_O and Ce(NO_3_)_3_·6H_2_O were bought from Tianjin Guangfu Fine Chemical Co. (China). Thirty percent of H_2_O_2_ was obtained from Beijing Chemical (China). Diclofenac (98%) was bought from Tokyo Chemical (Japan). HPLC grade acetonitrile was purchased from Fisher Scientific (USA).

### Synthesis of Catalysts

CuO was prepared by precipitation method (Ghasemi and Karimipour, 2018). One hundred and fifty milliliter NaOH solution (16.67 mol/L) was slowly injected into 150 mL of Cu(NO_3_)_2_ solution (0.67 mol/L). The mixture was stirred at ~1,000 rpm at a constant temperature of 80°C for 3 h. The precipitate was separated by centrifugation, in which the solution was centrifuged for 20 min at 3,500 rpm. The precursor of CuO was dried for 3 h at 105°C and then calcined for 3 h at 700°C. The CuO powder was obtained after grinding.

CeO_2_ was prepared by precipitation method (Zhao et al., 2014) and CuO/CeO_2_ was prepared by ultrasonic impregnation method. As such, 2 g prepared CeO_2_ powder was added to 20 mL 1 mol/L Cu(NO_3_)_2_ solution and impregnated for 30 min under 0.5 W/cm^3^ ultrasound, and then the filtered solid particulars were calcined for 2 h at 450°C in muffle furnace to obtain CuO/CeO_2_.

### Characterization of CuO/CeO_2_

The porosity and specific surface area of CuO/CeO_2_ were characterized by N_2_ adsorption-desorption test using 3H-2000PS2, BeiShiDe Instrument-S&T Company. CuO/CeO_2_ morphology was recorded by SEM with energy dispersive spectroscopy (EDS) (Hitachi S 4700 SEM analyzer). The XRD patterns were carried out by Rigaku D/maxrc diffractometer. The HRTEM measurement was investigated by Thermo Scientific Dionex UltiMate 3000 instrument. The oxidation state of CuO/CeO_2_ was characterized by XPS analysis (EScalab250Xi spectrometer), and the binding energies were calibrated by C 1s peak at 284.8 eV.

### Diclofenac Removal Experiments

The operation processes of reactions were as follows: 50 mL diclofenac solution (20 mg/L) was added to a 150 mL beaker and adjusted to the desired pH using 1 mol/L NaOH and H_2_SO_4_ solutions. After that, certain dosages of CuO/CeO_2_ and H_2_O_2_ were poured into the solution under continuous magnetic stirring. Finally, a small sample of the mixture was taken out by syringe at certain times and filtered by a 0.45 μm membrane filter. The filtered solution was measured by HPLC. The experiments were done in triplicate.

The diclofenac concentration in the experiment was analyzed by Thermo Scientific Dionex UltiMate 3000 liquid chromatographt with a C18 column. The operation parameters were as follows: the flowing phases were 70% acetonitrile and 30% acetic acid solution (volume fraction was 0.2%), the flow rate was 1 mL/min, the injection volume was 10 μL, and the detection wavelength was 275 nm. The leached metal ions were detected by inductively coupled plasma optical emission spectrometer (ICP-OES) (IRIS Intrepid II XSP, ThermoFisher).

## Results and Discussion

### Catalyst Characterization

#### N_2_ Adsorption-Desorption Analysis

**Figure 1:**
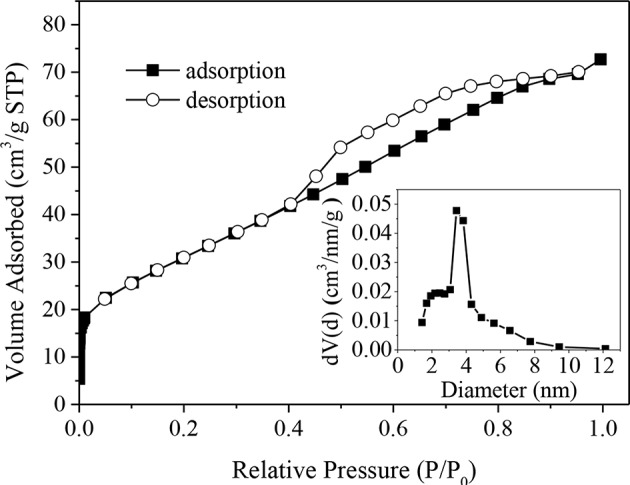
N_2_ adsorption-desorption isotherm and pore size distribution of CuO/CeO_2_.

Figure 1 shows the N_2_ adsorption-desorption isotherm of CuO-CeO_2_, which was typical type-IV isotherm with a H1-type hysteresis loop. This implied that mesoporous structure existed in the obtained CuO/CeO_2_. The pore diameter distribution of CuO/CeO_2_ was calculated from the adsorption branch and suggested the existence of mesopores at ~10 nm with the average diameter 3.9 nm (the embedded diagram of Figure 1). Table 1 summarized the basic structural parameters of CuO, CeO_2_, and CuO/CeO_2_. The specific surface area of CuO/CeO_2_ reached 114.8 m^2^/g which was higher than that of CuO and CeO_2_, and was also much higher than that of other CuO-containing catalysts such as CuO/rGO (56.1 m^2^/g) (Du et al., 2019) and CuO/Ti_6_O_13_ (6.93 m^2^/g) (Sehati and Entezari, 2017). The high specific surface area allowed the rapid electron transfer (Prathap et al., 2012) and would benefit the catalytic performance of CuO/CeO_2_. These results indicated that ultrasonic preparation might facilitate catalytic reaction by changing the surface structure of the catalyst. For one thing, the mechanical effect of ultrasound could cut CeO_2_ into smaller particles increasing specific surface area. Furthermore, the ultrasonic cavitation effect made CuO more uniformly distributed on the surface of CeO_2_. The decreases of pore volume and pore diameter after ultrasonic impregnation of CeO_2_ (Table 1) exactly indicated that CuO was indeed loaded on CeO_2_.

**Table 1:** Basic structural parameters of CuO, CeO_2_, and CuO/CeO_2_.

**Catalyst**	**Surface area (m^**2**^/g)**	**Pore volume (cm^**3**^/g)**	**Average pore diameter (nm)**
CuO	0.5	1.9 × 10^−3^	14.2
CeO_2_	78.5	1.9 × 10^−1^	9.4
CuO/CeO_2_	114.8	1.1 × 10^−1^	3.9

#### SEM Analysis

**Figure 2:**
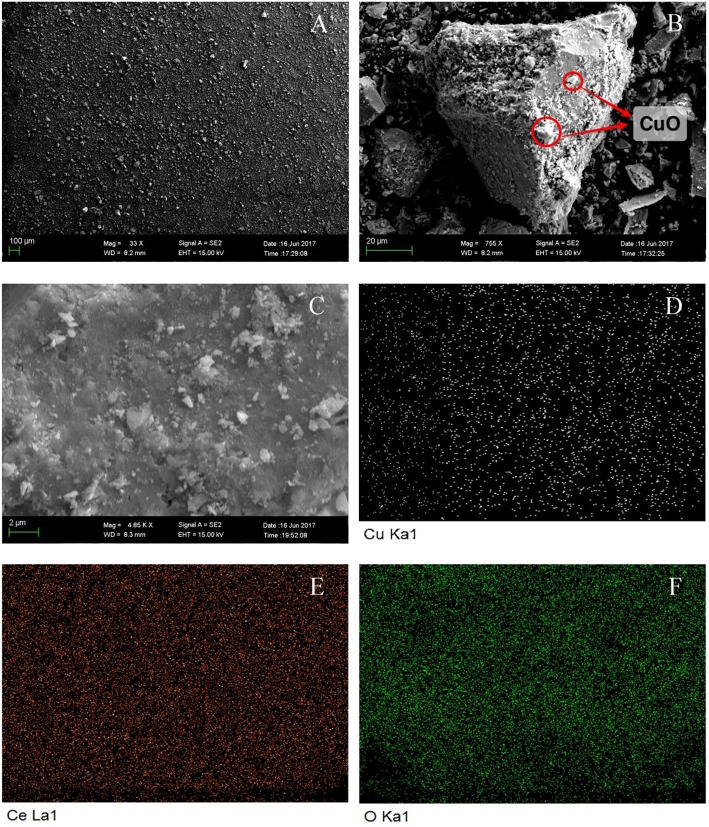
SEM analysis of CuO/CeO_2_: **(A)** general view, **(B)** detailed image, **(C)** mapping region, **(D)** Cu mapping image, **(E)** Ce mapping image, and **(F)** O mapping image.

SEM was used to characterize the morphology of CuO/CeO_2_, and the results were shown in Figure 2. Figure 2A shows an overview of CuO/CeO_2_ in which the size of particles was uniform. CuO was finely loaded on CeO_2_ (Figure 2B). Element mapping results of Cu, Ce, and O (Figures 2D–F) indicated that the three elements were all well-dispersed in the catalyst. To investigate how ultrasound affected the structure of catalyst, Mahdiani et al. (2018) prepared PbFe_12_O_19_ with and without ultrasound, and found that ultrasound could decompress the large structures and form fine and homogeneous structures. Thus, the fine dispersion of elements and uniform size of CuO/CeO_2_ benefitted from the cavitation effect of ultrasound which created an intense environment in the reaction mixture (Shende et al., 2018). Moreover, Hočevar et al. (2000) showed that the high dispersion of Cu on CeO_2_ had positive effect on the activity and selectivity of copper cerium oxide. The Cu, Ce, and O atomic percentages confirmed by EDS were 10.56, 18.07, and 71.37%, respectively. The lower content of Cu showed that the dispersion of Cu was relatively sparse.

#### XRD Analysis

**Figure 3:**
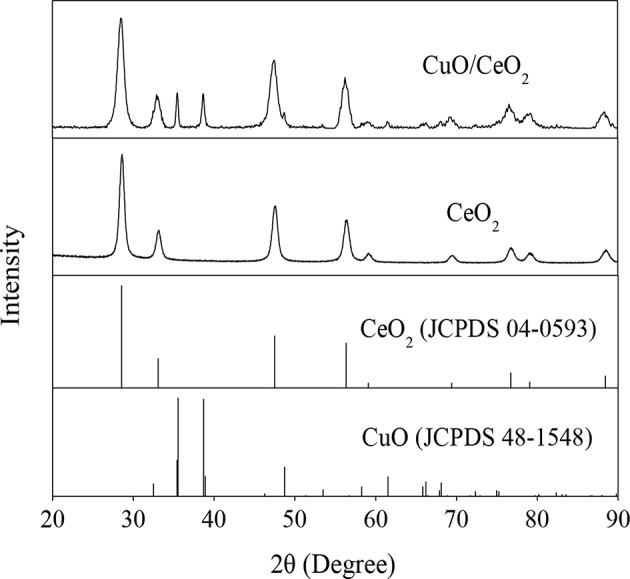
XRD pattern of CuO/CeO_2_.

XRD analysis was applied to identify the component and crystallography of the catalyst. The results were shown in Figure 3. The diffraction peaks at 28.46°, 32.9°, 47.44°, 56.24°, 76.260°, and 79.22° could be attributed to the cubic fluorite CeO_2_ (JCPDS No. 48-1548), which are characteristics of the (111), (200), (220), (311), (400), and (331) crystal faces. There were also characteristic diffraction peaks owing to CuO crystal, and 35.46°, 38.66° were assigned to the (002) and (111) faces (JCPDS No. 04-0593). The characteristic diffraction peaks of CeO_2_ in CuO/CeO_2_ were slightly broadened compared with that in CeO_2_. This might be associated with the incorporation of Cu^2+^ ions with a smaller ionic radius (0.73 Å) compared with Ce^4+^ (0.97 Å) (Cau et al., 2014). These phenomena indicated that lattice constriction occurred in CuO/CeO_2_.

#### TEM and HRTEM Analyses

**Figure 4:**
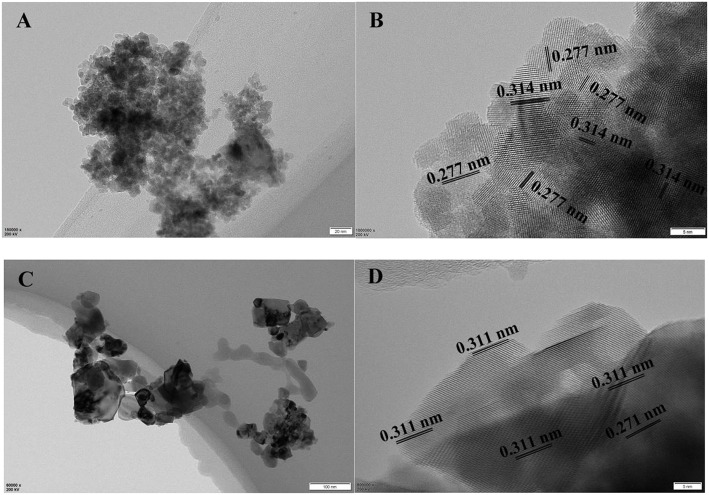
**(A)** TEM and **(B)** HRTEM images of CuO/CeO_2_, **(C)** TEM and **(D)** HRTEM images of CeO_2_.

The TEM images of CuO/CeO_2_ and CeO_2_ were shown in Figures 4A,C. The particle size of CuO/CeO_2_ was uniform, and was generally smaller than that of CeO_2_, which was a favorable result of ultrasonic impregnation method and was good for catalytic reaction. The lattice fringes of CuO/CeO_2_ were measured at 2.77 and 3.14 Å (Figure 4B), corresponding to (200) and (111) crystal faces. Compared with the two crystal faces of CeO_2_ (Figure 4D), the lattice fringe spacing in CuO/CeO_2_ had a slight increase. This phenomenon might be caused by some Cu atoms doped into CeO_2_ structure_._ CuO/CeO_2_ had lattice constriction and generated a solid solution structure. The XRD analysis showed similar results.

The crystal face (111) of CeO_2_ was the most stable crystal face in CeO_2_ for its minimal surface energy. Thus, the stability of the catalyst would be enhanced with the increase of exposed crystal face (111). The catalytic activity could be improved by the crystal face (200) for its high surface energy. Moreover, oxygen vacancies were more favored to form on unstable face (200) than on (111). Thus, exposed (200) and (111) faces were beneficial to the catalytic performance and stability of CuO/CeO_2_.

#### Raman Analysis

**Figure 5:**
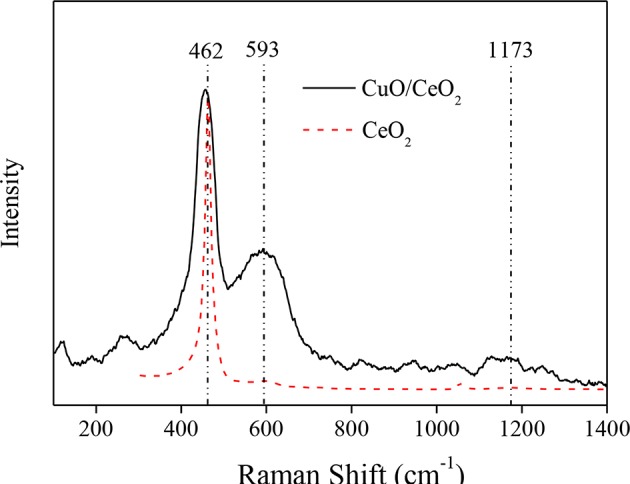
Raman spectra of CuO/CeO_2_ and CeO_2_.

To investigate the effect of CuO doping on the quantities of oxygen vacancies over CuO/CeO_2_, CuO/CeO_2_, and CeO_2_ were both characterized by Raman spectra. As shown in Figure 5, a strong peak at 462 cm^−1^ was observed, which was attributed to the vibration mode of the F2 g symmetry in cubic fluorite CeO_2_ lattice. This peak of CuO/CeO_2_ presented a red shift compared to pure CeO_2_ because of the reduction in the lattice parameter as a result of shorter M-O bond length (Cau et al., 2014). The weak band at 1,172 cm^−1^ was assigned to the second-order phonon mode of fluorite structure. The band at 593 cm^−1^ was attributed to oxygen vacancies in CeO_2_. With Cu doping into CeO_2_, more oxygen vacancies were generated, which would promote the catalytic property of CuO/CeO_2_.

#### XPS Analysis

**Figure 6:**
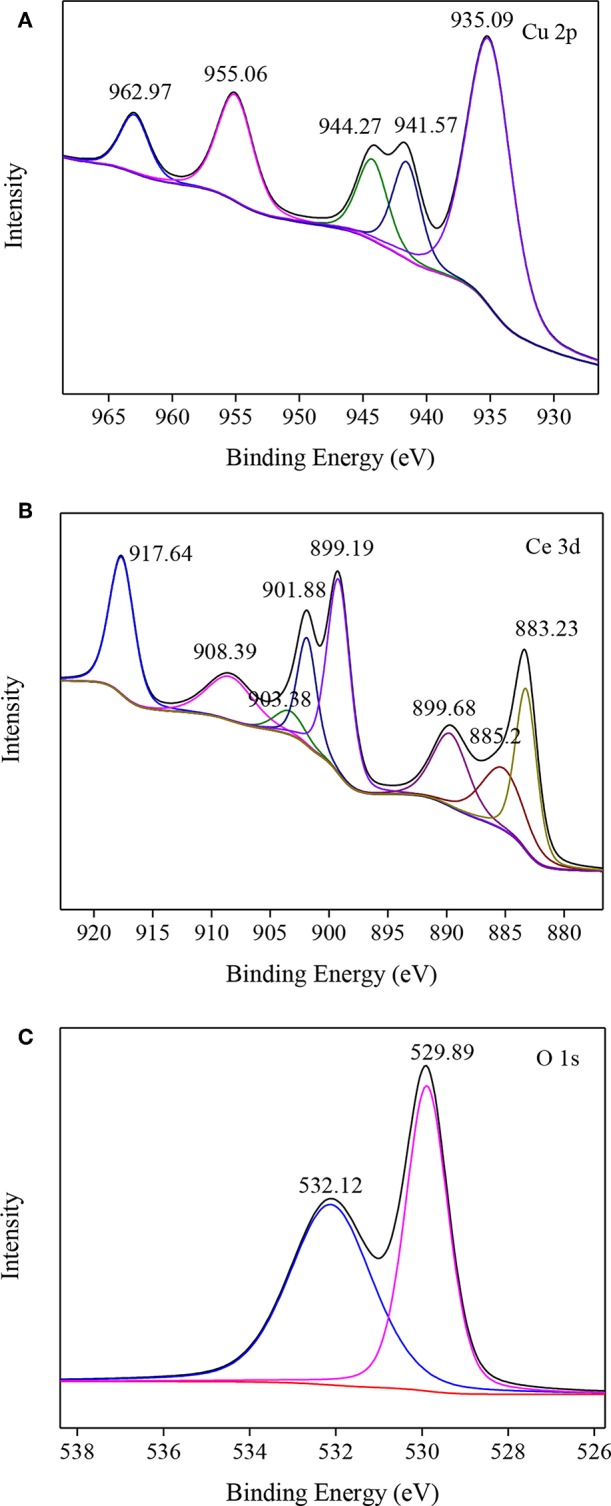
XPS spectra of **(A)** Cu 2p, **(B)** Ce 3d, and **(C)** O 1s in CuO/CeO_2_.

The valence states of Cu, Ce, and O in CuO/CeO_2_ were investigated by XPS analysis. Figure 6A shows the XPS spectrum of Cu 2p of CuO/CeO_2_. The peak at 935.09 eV was the main peak of Cu 2p_3/2_, which was related to CuO (Jawad et al., 2018). CuO had a satellite peak accompanied by the main peak, which was approximately 9 eV higher binding energy than the main peak (Zeng et al., 2013). Thus, 944.27 eV was the satellite peak of CuO.

The XPS spectrum of Ce 3d is shown in Figure 6B. The four main 3d_5/2_ features appeared at 883.23, 885.2, 889.68, and 899.19 eV, while the 3d_3/2_ features appeared at 901.68, 903.38, 908.39, and 917.64 eV. Among them, the peaks at 883.23 and 901.68 eV were assigned to characteristic of Ce^3+^, indicating that there were two valence states of Ce (+3 and +4) existed in CuO/CeO_2_.

The O 1s XPS spectrum was shown in Figure 6C. O 1s existed in two groups, O^2−^ and OH^−^, which were fitted with peaks at 529.89 and 532.12 eV, respectively. O^2−^ groups represented the lattice oxygen (O_latt_) in metal oxides, generating from CuO and CeO_2_. The peak at 532.12 eV was the characteristic of adsorbed oxygen species or surface OH species. The chemical adsorbed oxygen (O_ads_) on the surface of CuO/CeO_2_ might be transformed from O_latt_ through oxygen vacancies, which was a reactive oxygen specie to attack organics (Chong et al., 2017). This kind of conversion demonstrated that O_latt_ species participated in the degradation process of diclofenac, which was consistent with the study by Sedmak et al. (2003). Furthermore, XPS results indicated that CuO doped in CeO_2_, which could improve the formation of O_latt_ in CuO/CeO_2_ (Zou et al., 2011).

### Diclofenac Removal in CuO/CeO_2_-H_2_O_2_ Fenton-Like System

**Figure 7:**
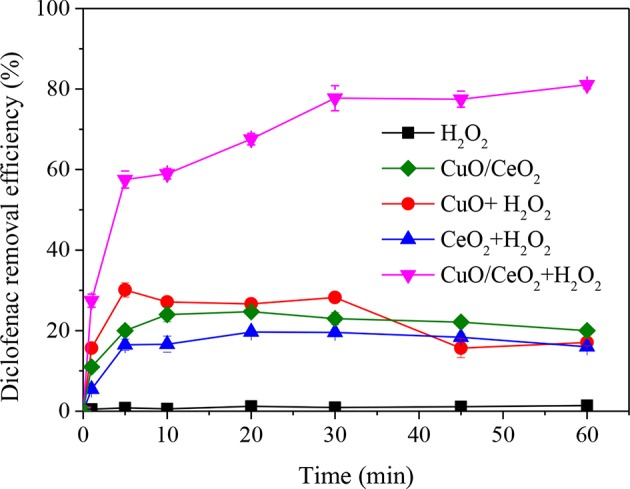
Removal efficiency of diclofenac in different systems. Reaction conditions: pH = 5, H_2_O_2_ = 200 mg/L, catalyst = 1 g/L.

The comparison of diclofenac removal efficiency by H_2_O_2_, CuO/CeO_2_, CuO-H_2_O_2_, CeO_2_-H_2_O_2_, and CuO/CeO_2_-H_2_O_2_ was shown in Figure 7. Single H_2_O_2_ and single CuO/CeO_2_ could hardly remove diclofenac, the same for CuO-H_2_O_2_ and CeO_2_-H_2_O_2_ system. However, CuO/CeO_2_ could effectively catalyze H_2_O_2_ to remove diclofenac. The highest removal efficiency was 81.05, 30.01, and 19.67% for CuO/CeO_2_-H_2_O_2_, CuO-H_2_O_2_ and CeO_2_-H_2_O_2_ processes, respectively. Compared with CuO-H_2_O_2_ and CeO_2_-H_2_O_2_ systems, diclofenac removal efficiency after 60 min in CuO/CeO_2_-H_2_O_2_ system was increased by 63.99 and 65.05%, respectively.

Clearly, CuO/CeO_2_-H_2_O_2_ system had higher diclofenac removal than the sum of CuO-H_2_O_2_ system and CeO_2_-H_2_O_2_ system, showing a synergistic effect between CuO and CeO_2_. The synergistic effect might be caused by two reasons. Firstly, more oxygen vacancies were formed for Cu doped in CeO_2_, which was shown in the above characterization analyses. Lu et al. (2011) found that the formation energy of oxygen vacancy in Cu-doped ceria was lower than bare ceria, demonstrating that Cu dopant could serve as a seed for the formation of oxygen vacancies. Secondly, there were electronic interactions between metal and the support, i.e., the facilitation of redox interactions between Cu^2+^/Cu^+^ and Ce^4+^/Ce^3+^ redox couples, which would favor the CuO reduction (Konsolakis, 2016). Studies (Szabová et al., 2010, 2013) also reported that Cu doped on CeO_2_ surface accompanied electron transfer process between Cu and neighboring Ce^4+^, generating more Ce^3+^.

### Optimization of Operation Parameters for Diclofenac Removal

**Figure 8:**
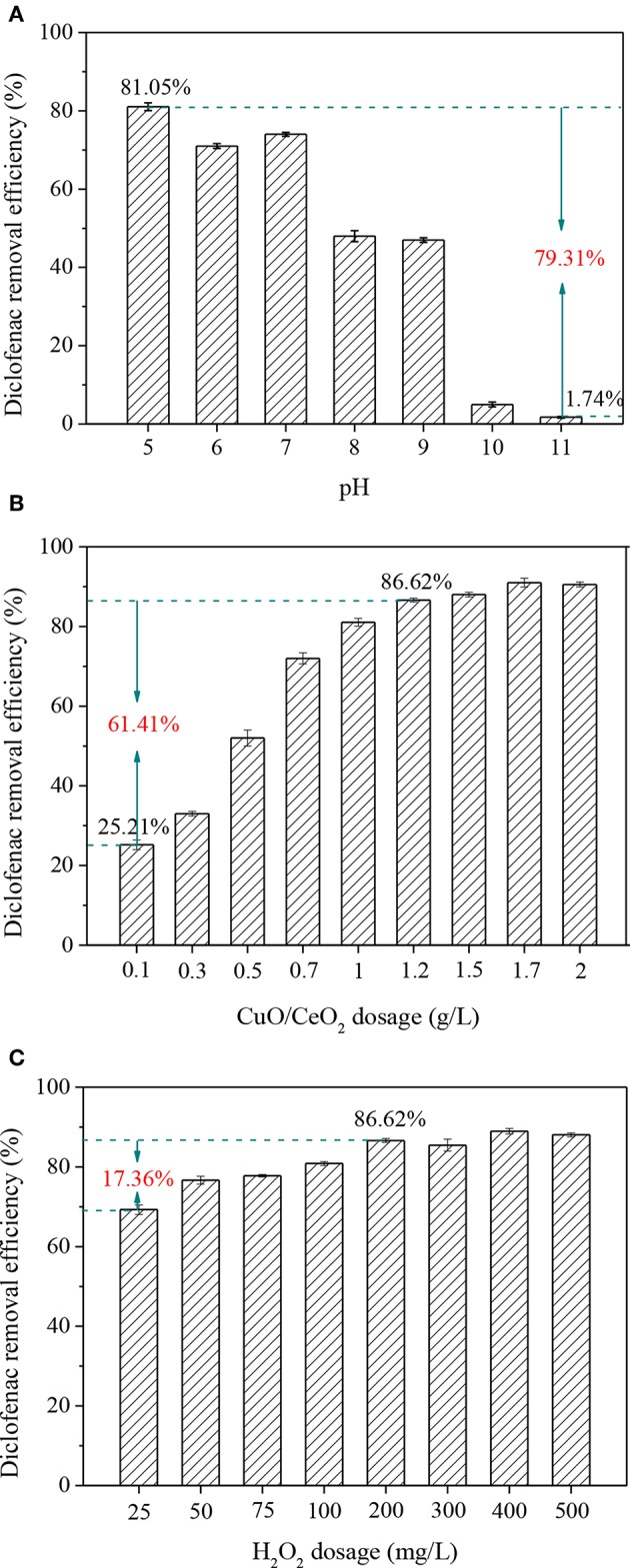
Influencing factors of diclofenac removal in CuO/CeO_2_-H_2_O_2_ system: **(A)** pH, **(B)** CuO/CeO_2_ dosage, **(C)** H_2_O_2_ dosage. Reaction conditions: **(A)** H_2_O_2_ = 200 mg/L, catalyst = 1 g/L; **(B)** pH = 5, H_2_O_2_ = 200 mg/L; **(C)** pH = 5, catalyst = 1.2 g/L.

pH is an important parameter in AOPs. An advantage of Fenton-like process over Fenton reaction is avoiding of too acidic condition (pH < 3.5). As known, diclofenac was more likely to dissociate to ionic structure when the pH was higher than its pK_a_ (4.2), which was favorable to its adsorption and degradation. Thus, the effect of pH above 5 was investigated. As shown in Figure 8A, pH had a significant effect on diclofenac removal in CuO/CeO_2_-H_2_O_2_ system, which decreased by 79.31% when the pH changed from 5 to 11. The result might attribute to two reasons: (1) the oxidation potential of ·OH decreased, and more H_2_O_2_ decomposed to O_2_ and H_2_O at high pH value; (2) deprotonation of the catalyst gradually increased, which was unfavorable to the electrostatic attraction between diclofenac and CuO/CeO_2_ (Hassani et al., 2018).

Different dosages of CuO/CeO_2_ were added to the Fenton-like system. Figure 8B shows that the diclofenac removal efficiency was improved from 25.21 to 86.62% when the catalyst dosage increased from 0.1 to 1.2 g/L. The active sites were mostly on the surface of the catalyst, so more catalyst meant that more active sites exposed. But when the active sites were adequate in CuO/CeO_2_-H_2_O_2_ system, further addition of catalyst would bring little improvement. Thus, the optimum CuO/CeO_2_ dosage was 1.2 g/L.

The effect of H_2_O_2_ on diclofenac removal was studied within the dosage of 25–500 mg/L. Figure 8C shows that with the continuous increase of H_2_O_2_ dosage, the removal efficiency of diclofenac reached 86.62% at 200 mg/L H_2_O_2_, then the reaction tended to stabilize. Excessive H_2_O_2_ reacted with ·OH, thus go against the diclofenac removal (Hassani et al., 2018). Taking into account the removal efficiency and cost, 200 mg/L was considered as the optimum dosage of H_2_O_2_.

Lee et al. (2014) found only about 24% diclofenac could be removed in 60 min by Cu(II)/H_2_O_2_ system. Xu et al. (2016) used Cu-doped α-FeOOH as Fenton-like catalyst to degrade diclofenac, <50% diclofenac was removed in 60 min. Zhou et al. (2018) found 80% diclofenac was removed in a magnetic field enhanced Fe^0^/EDTA Fenton-like system. In comparsion, CuO/CeO_2_ was a high-efficiency Fenton-like catalyst for diclofenac removal. Moreover, leaching test showed that there was almost no Ce leached (<0.05 mg/L) under the above optimal reaction conditions. The leached Cu concentration in the solution was 1.7 mg/L, which met the wastewater discharge standard of China (Cu < 2 mg L^−1^) (Wang et al., 2016) and was only 9‰ of the catalyst dosage. Besides, the removal of diclofenac still reached 70.12% in the third run of CuO/CeO_2_-H_2_O_2_ system. These results showed a good stability and reusability of the CuO/CeO_2_ catalyst.

### Potential Mechanism of Diclofenac Degradation in CuO/CeO_2_-H_2_O_2_ System

**Figure 9:**
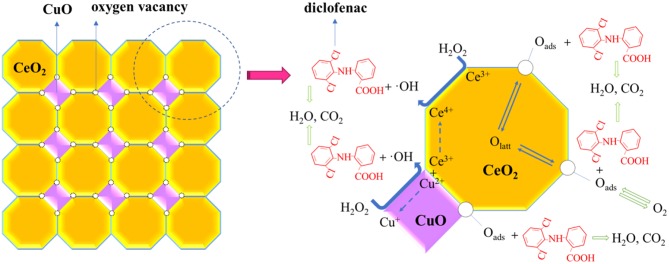
Potential mechanisms of diclofenac degradation in CuO/CeO_2_-H_2_O_2_ system.

Potential mechanism of diclofenac degradation in CuO/CeO_2_-H_2_O_2_ system was proposed in Figure 9. CuO could decompose H_2_O_2_ to form highly active ·OH (Drijvers et al., 1999), and the reaction was represented by Equations (1) and (2) (Yamaguchi et al., 2018). ·OH radical could also be produced by Ce^3+^ with H_2_O_2_, which was achieved by the division of O-O bond of H_2_O_2_, as shown in Equation (3) (Chong et al., 2017). Moreover, the synergistic copper and ceria interaction (Equation 4) facilitated the redox cycles of Cu^2+^/Cu^+^ and further was conducive to ·OH generation (Konsolakis, 2016). Sehati and Entezari (2017) found that ·OH on the catalyst surface (·OH_ads_) was the major reactive species, while ·OH in the solution had little effect on pollutant removal in Fenton-like system. During diclofenac degradation, diclofenac molecules were adsorbed on the surface of the catalyst, meanwhile, H_2_O_2_ was decomposed to ·OH by Cu and Ce species on the catalyst surface. Then, the adsorbed diclofenac molecules reacted with ·OH_ads_ and transformed into smaller molecules (intermediate products), H_2_O or CO_2_. The process continued until the complete decomposition of diclofenac was achieved (Ziylan et al., 2013).

(1) $$\begin{array}{r} Cu^{2+}\;+\;H_{2}O_{2}\;\to \;Cu^{+}\;+\;\cdot OOH\;+\;H^{+} \end{array}$$

(2) $$\begin{array}{r} Cu^{+}\;+\;H_{2}O_{2}\;\to \;Cu^{2+}\;+\;\cdot OH\;+\;OH^{-} \end{array}$$

(3) $$\begin{array}{r} Ce^{3+}\;+\;H_{2}O_{2}\;+\;H^{+}\;\to \;Ce^{4+}\;+\;\cdot OH\;+\;H_{2}O \end{array}$$

(4) $$\begin{array}{r} Cu^{2+}\;+\;Ce^{3+}\;\to \;Cu^{+}\;+\;Ce^{4+} \end{array}$$

More oxygen vacancies were generated by Cu doped into CeO_2_, which was proven by the Raman spectra (Figure 5). The oxygen storage capacity of a ceria-based catalyst benefited from oxygen vacancies (Soler et al., 2016). In the degradation process, the adsorbed O_2_ on the surface of CuO/CeO_2_ and bulk O_latt_ could replenish oxygen vacancies by diffusion from the surface and inner of the catalyst (Balcaen et al., 2010), then transformed to active oxygen (O_ads_) accompanied by valence state transformation of CeO_2_ and CuO (Ce^3+^/Ce^4+^ and Cu^+^/Cu^2+^) (Dong et al., 2019). O_ads_ could also participate in the oxidation of diclofenac (Xian et al., 2019). Besides, large specific surface area and high porosity of ultrasonically prepared CuO/CeO_2_ catalyst allowed rapid electron transfer, which improved the catalytic property (Prathap et al., 2012).

## Conclusions

An efficient heterogeneous Fenton-like catalyst CuO/CeO_2_ was synthesized by ultrasonic impregnation method and used to remove diclofenac from water. The prepared CuO/CeO_2_ had large specific surface area, high porosity and fine elements dispersion. CuO and CeO_2_ crystals coexisted in CuO/CeO_2_ with a lattice constriction. HRTEM analysis demonstrated that the main exposed crystal faces of CeO_2_ contained (200) face which readily formed oxygen vacancies and improved the catalytic property of CuO/CeO_2_. Oxygen vacancies in CeO_2_ were increased by Cu doping. The optimal operating conditions of CuO/CeO_2_-H_2_O_2_ system were pH = 5, CuO/CeO_2_ dosage = 1.2 g/L, and H_2_O_2_ dosage = 200 mg/L, with 86.62% diclofenac removal. The synergistic effect between CuO and CeO_2_ on diclofenac removal might be caused by more oxygen vacancies generation and electronic interactions between Cu and Ce species in CuO/CeO_2_. The degradation of diclofenac was mainly by ·OH and adsorbed oxygen species which were enhanced by oxygen vacancies.

## Data Availability Statement

All datasets generated for this study are included in the article/supplementary material.

## Author Contributions

GZ, GX, and JZ designed the experiments. GX, NZ, and JL performed the experiments. GZ and GX wrote the paper. GZ, GX, NZ, and JZ discussed the results and analyzed the data.

### Conflict of Interest

The authors declare that the research was conducted in the absence of any commercial or financial relationships that could be construed as a potential conflict of interest.
